# Educational Anomaly Analytics: Features, Methods, and Challenges

**DOI:** 10.3389/fdata.2021.811840

**Published:** 2022-01-14

**Authors:** Teng Guo, Xiaomei Bai, Xue Tian, Selena Firmin, Feng Xia

**Affiliations:** ^1^School of Software, Dalian University of Technology, Dalian, China; ^2^Computing Center, Anshan Normal University, Anshan, China; ^3^School of Arts, Law and Education, University of Tasmania, Launceston, TAS, Australia; ^4^School of Engineering, IT and Physical Sciences, Federation University Australia, Ballarat, VIC, Australia

**Keywords:** anomaly analytics, educational big data, machine learning, data science, anomaly detection

## Abstract

Anomalies in education affect the personal careers of students and universities' retention rates. Understanding the laws behind educational anomalies promotes the development of individual students and improves the overall quality of education. However, the inaccessibility of educational data hinders the development of the field. Previous research in this field used questionnaires, which are time- and cost-consuming and hardly applicable to large-scale student cohorts. With the popularity of educational management systems and the rise of online education during the prevalence of COVID-19, a large amount of educational data is available online and offline, providing an unprecedented opportunity to explore educational anomalies from a data-driven perspective. As an emerging field, educational anomaly analytics rapidly attracts scholars from a variety of fields, including education, psychology, sociology, and computer science. This paper intends to provide a comprehensive review of data-driven analytics of educational anomalies from a methodological standpoint. We focus on the following five types of research that received the most attention: course failure prediction, dropout prediction, mental health problems detection, prediction of difficulty in graduation, and prediction of difficulty in employment. Then, we discuss the challenges of current related research. This study aims to provide references for educational policymaking while promoting the development of educational anomaly analytics as a growing field.

## 1. Introduction

Education plays an important role in human development. However, the process of education is not always smooth. Unexpected phenomena occur from time to time, leading to adverse consequences. For example, students who drop out of university face social stigma, fewer job opportunities, lower salaries, and a higher probability of involvement with the criminal justice system (Amos, 2008). Students who suffer from depression may exhibit extreme behaviours such as self-harm, or even suicide (Jasso-Medrano and Lopez-Rosales, 2018). Although universities set up institutions to help them, not everyone is proactive in seeking help. Exploring and understanding the laws behind educational anomalies enables educational institutions (such as high schools and universities) to be more proactive in helping students succeed in their personal lives and careers. As a result, this field attracts many scholars from various fields, such as computing, psychology, and sociology.

Researchers cannot conduct scientific inquiry without high-quality data. The essence of education is knowledge delivery, and its process is challenging to quantify and record, causing the inaccessibility of educational data. Previous research in this field is based on questionnaires, which are time- and cost-consuming and hardly applicable to large-scale student cohorts. Computer technology has brought significant change to the education field in recent years. Due to the popularity of learning management systems (LMS), data from many traditional educational institutions (e.g., high schools and universities) is collected. Meanwhile, the epidemic of COVID-19 has led to teaching being undertaken remotely and on digital platforms, which has extensively promoted the growth of online education. This process generates a lot of data for online education (Liu et al., 2021; Yu et al., 2021b). These changes have contributed significantly to the development of big data technologies in education and provide a unique opportunity for educational anomaly analytics (Hou et al., 2019; Al-Doulat et al., 2020; AlKhuzaey et al., 2021; Ren et al., 2021).

Currently, a large number of researchers have concentrated their efforts on such an emerging area with the technology of big data, yielding impressive results (Bai et al., 2020; Hou et al., 2020; Zhang et al., 2020; Xia et al., 2021). A systematic review is urgently needed to sort out the results and challenges of current research and to provide references for educational policymaking and subsequent research. Several scholars have already published review papers in related fields. Moreno-Marcos et al. (2018) give a targeted analysis of the predictions in MOOC, especially the dropout predictions, through a systematic literature review. Hellas et al. (2018) present a systematic literature review of works predicting students' performance in computing courses. Refer to Table 1 for the rest of the related survey papers. They, however, focus on the discussion of individual anomalies rather than a systematic comparative analysis of all educational anomalies. Meanwhile, most current reviews focus on questionnaire-based correlation analysis of variables rather than data-driven research based on machine learning techniques.

**Table 1:** Survey papers addressing educational anomalies.

Moreno-Marcos et al. (2018)	Marcos et al. give a targeted analysis of the predictions in massive open online course (MOOC), especially the dropout predictions, through a systematic literature review.
Hellas et al. (2018)	Hellas et al. present a systematic literature review of works predicting students' performance in computing courses, by analysing the results of 357 papers.
Alturki et al. (2020)	Alturki et al. summarise the relevant features (mainly including historical performance and demographic features) and the advantages and disadvantages of the prediction algorithm.
Khan and Ghosh (2020)	Khan et al. present a systematic review of educational leadership and policy (EDM) studies on student performance in classroom learning.
Rastrollo-Guerrero et al. (2020)	Rastrollo-Guerrero et al. analyse the application of machine learning techniques to education-related predictions, including predictions of academic performance and activities.
Alban and Mauricio (2019)	Alban et al. provide a detailed list of all the features and methods mentioned in the dropout prediction study and analyses them in detail.
Mduma et al. (2019)	Mduma et al. analyse and summarise machine learning techniques used in dropout prediction.
Liz-Domínguez et al. (2019)	Liz-Dominguez et al. provide a detailed review of prediction algorithms applied to higher education, with special attention to early warning systems.

**Figure 1:**
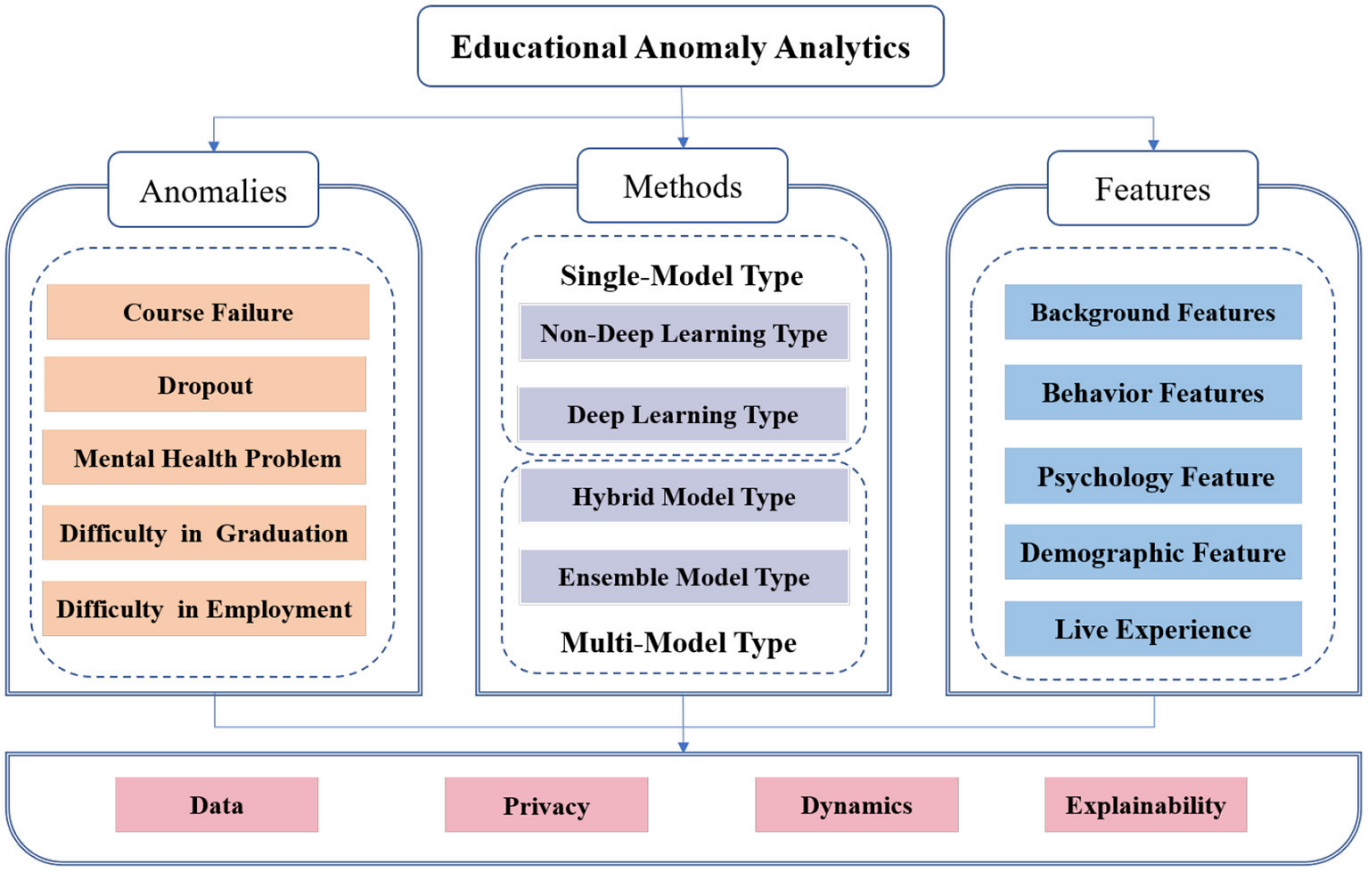
Framework of educational anomaly analytics.

Against this background, we intend to conduct a systematic overview of data-driven educational anomalies analytics to fill the gap mentioned above. In this paper, we innovatively introduce the concept of educational anomalies, that is, the behaviour or phenomenon that interferes with a student's campus life, studies, degree attainment, and employment (Barnett, 1990; Sue et al., 2015; Zhang et al., 2021). Note that the anomalies defined in this paper are mainly negative issues that affect physical and mental health and academic development. Neutral issues, such as entrepreneurial intention or learning habits, for example, are not included in the scope of our study. We focus on the following five types of research that received the most attention, including course failure prediction, dropout prediction, mental health problems detection, prediction of difficulty in graduation, and prediction of difficulty in employment (shown in Figure 1). First, we introduce the classification of methods and features in related works. Second, for each type of educational anomaly, we summarise the relevant works from the perspective of features and methods. In addition, we summarise the current challenges in this area.

Our contributions can be outlined as follows:

- To the best of our knowledge, this study is the first to conduct a systematic overview of data-driven educational anomaly analytics.
- We present an innovative classification of features and algorithms in the relevant fields, with a comprehensive comparison and targeted discussion.
- Our conclusions provide references for educational policymaking while promoting the development of educational anomaly analytics.

This paper is organised as follows. In section 2, we describe the methodology in this paper. In section 3, we analyse works related to the prediction of student course failure. Next, works predicting student dropouts are analysed in section 4. In section 5, we analyse works that detect students with mental health problems. In section 6, works for the prediction of difficulty in graduation are summarised. Next, works for the prediction of difficulty in employment are summarised in section 7. Then, we present the challenges of current research in the field in section 8. Finally, we present a conclusion of our work in section 9.

## 2. Methodology

### 2.1. Criteria for Paper Collection

To conduct a comprehensive survey of the latest trends of educational anomalies, we define a set of inclusion and exclusion criteria for paper collection shown as follows:

(1) The study is written in English.
(2) The study is published in a scientific journal, magazine, book, book chapter, conference, or workshop.
(3) The study is published from 2016 to 2021.
(4) The study is excluded if not fully focused on the educational anomalies.
(5) The study is based on the data-driven method.

We mainly search for the following keywords: academic performance prediction, dropout prediction, mental health problems detection, graduation prediction, and employment prediction on Google Scholar and Microsoft Academic. Note that, for some special cases, we carry out a further targeted search. For example, in the collection of papers on college students' mental health, we find that depression attracted a large number of scholars' attention. Therefore, we would do further article collection based on keywords like college student depression prediction. We first find some relevant references published in important journals and conferences. Secondly, based on these references, we further lookup which references are cited by these existing references one by one, and at the same time, we look up which references are cited in the current literature. According to this method, we search for more than 300 related papers. According to the above criteria, we then manually screen these references one by one for our research. Finally, we retain 134 papers that are the most relevant.

### 2.2. Taxonomy

In this section, we describe the details of the classification of features and methods in this paper.

#### 2.2.1. Classification of Features

Due to the rapid development of Information and Communication Technology (ICT) in the last decade, a large amount of educational data has been collected, leading to a more diverse range of features being used to predict or detect educational anomalies. By summarising the relevant work, we have grouped all the features into the following five categories:

*Background Feature*: This type of feature contains two types of sub-features: historical academic performance and demographic features. This type of feature reflects static background information about the student. This type of feature is frequently in traditional research.

*Behaviour Feature*: Given the development of information technology in the last decade, increasingly computer-based and information science technologies are being used in education, leading to various data generated in the process of campus life (including the living process and studying process), being recorded, and these data record all student behaviours in learning and life. If the background feature is a static feature for students, then the behaviour feature is dynamic. This data brings us new perspectives on portraying students, which has become a hot topic in the related field in the past few years. Generally, we divide students' behaviours into learning behaviours related to learning and daily behaviours that are not associated with learning.

*Psychological Feature*: Psychological features, mainly quantify the mental state of students, contain data related to historical, psychological questionnaire tests.

*Live Experience Feature*: Live experience features, including particular life experiences, are mainly used for psychologically related detections.

#### 2.2.2. Classification of Methods

With the advent of a data-driven fourth research paradigm, scholars are scrambling to introduce machine learning-related methods to predict or detect educational anomalies. By summarising the relevant works, we find that the number of models used in the experimental design was closely related to the experimental intent. In this case, we have divided the relevant work into the following two broad categories: single-model type and multiple-model type, to provide a more precise summary of the current research.

The single-model type includes two types shown as follows:

- Non-Deep learning type: Prediction or detection experiments are designed based on one single non-deep learning model.
- Deep learning type: Prediction or detection experiments are designed based on deep learning models.

Since the underlying principles are different, this paper divides the models into deep learning models and non-deep learning models. The multiple-model type is divided into two types, shown as follows:

- Hybrid model type: Multiple learning models are used to make predictions or detections independently and select the best one.
- Ensemble model type: Multiple learning models are integrated using bagging, boosting, and stacking methods to make predictions or detections.

Generally, the choice of the method implies a tendency of the work. Next, we describe the details of several types of work. First, the purpose of research categorised as non-deep learning type is to explore the correlation between variables. They often choose white box models with strong explainability, such as Linear Regression (shown as Equation 1) and Decision Tree based on Information Entropy (Equation 2) or Gini Index (Equation 3). This type of work rarely has methodological innovation, and its highlight lies in discovering relationships between variables.


(1) $$\begin{array}{l} Y=a+b\cdot X+e \end{array}$$

where *a* represents the intercept, *b* represents the slope of the line, and *e* is the error term.


(2) $$\begin{array}{l} \mathrm{Ent}\left(D\right)=-\overset{n}{\underset{i}{\sum }}p_{k}\log_{2}p_{k} \end{array}$$

where *D* represents the sample set and *p*_*k*_ represents the proportion of sample *k*.


(3) $$\begin{array}{l} \mathrm{Gini}\left(D\right)=\overset{\left|y\right|}{\underset{k=1}{\sum }}\underset{k^{\prime }\neq k}{\sum }p_{k}p_{k}^{\prime }=1-\overset{\left|y\right|}{\underset{k=1}{\sum }}p_{k}^{2} \end{array}$$

On the contrary, works of deep learning type pursue the final prediction or detection performance rather than explainability. This type of work predicts educational anomalies based on neural networks and back-propagation-related theories, which is currently a popular type of research. Similarly, the purpose of work belonging to the multiple-model type is to pursue higher prediction or detection performance. The models that often appear in this type of research are non-deep learning models. The models that often appear in this type of research are non-deep learning models, including the white-box models mentioned above and machine learning models, such as support vector machine (SVM) (Equation 4) and Bayesian-related algorithm (Equation 5).


(4) $$\{\begin{array}{l} \min\frac{\|w{\|}^{2}}{2}\\ \text{ s. t. }y_{i}\left(wx_{i}+b\right)⩾1,\;i=1,2,\cdots ,l \end{array}$$

(5) $$\begin{array}{l} P\left(B_{i}|A\right)=\frac{P\left(B_{i}\right)P\left(A|B_{i}\right)}{\sum_{j=1}^{n}P\left(B_{j}\right)P\left(A|B_{j}\right)} \end{array}$$

Works of hybrid model type simply try the performance of different models one by one and choose the best ones. This kind of research has more application value than theoretical innovation. Works of ensemble model type use more scientific and practical means to combine all models to achieve better performance. For example, scholars obtain stronger classifiers by constructing linear combinations of basic classifiers (shown in Equations 6, 7)


(6) $$\begin{array}{l} f\left(x\right)=\overset{M}{\underset{m=1}{\sum }}\alpha_{m}G_{m}\left(x\right) \end{array}$$

(7) $$\begin{array}{l} G\left(x\right)=\mathrm{sign}\left(f\left(x\right)\right)=\mathrm{sign}\left(\overset{M}{\underset{m=1}{\sum }}\alpha_{m}G_{m}\left(x\right)\right) \end{array}$$

where *G*_*m*_(*x*) represent *m*th basic classifier and α_*m*_ is its weight.

In terms of algorithm performance, deep learning-related algorithms are generally better than other algorithms. But poor explainability is one of its undoubted shortcomings. More specific algorithm selection is related to the experimenter's intention, the size of the dataset, the dimension of the feature, and so on.

## 3. Course Failure Prediction

Course performance is the main criterion for quantifying a student. Predicting course failure contributes to the development of individual students and provides a reference for the design of course content and the evaluation of the teachers involved. Currently, predicting students' course failure attracts most of the attention in the relevant field. In this section, we review related works from the perspective of methods and features.

### 3.1. Features for Course Failure Prediction

Campus-life and various features are predictors of course failure. Characteristics are used to divide these into three categories: background feature (historical academic performance and demographic features), behaviour feature (online behaviour, offline behaviour, Internet access pattern, library record, and social pattern), and psychological data. The details are shown as follows.

#### 3.1.1. Background Features

Background features appear with a high frequency in studies of course failure prediction (Livieris et al., 2016; Hu et al., 2017; Tsiakmaki et al., 2018; Francis and Babu, 2019; Hassan et al., 2019; Hung et al., 2019; Yu et al., 2020). Livieris et al. (2016) present a new user-friendly decision support tool for predicting students' performance concerning the final examinations of a school year, and they choose demographic features and historical academic performance as features. Hu et al. (2017) focus on the course-specific model for academic performance prediction and also use age, race, gender, and high school GPA as features. Tsiakmaki et al. (2018) also design experiments to predict students with course failure based on their background features.

Scholars are keen on using background features for prediction for the following reasons: First, historical course performance is used as a feature because research has demonstrated a correlation between course performance over time (Voyer and Voyer, 2014; Hedefalk and Dribe, 2020). Essentially, this reflects the student's IQ and attitude toward learning, and these things do not change in a short time period. Second, numerous studies demonstrate that background features affect students' academic performance (Voyer and Voyer, 2014; Hedefalk and Dribe, 2020). These two features are relatively easy to collect because this type of information is stored in the learning management system (LMS).

It is worth acknowledging that background features have a good contribution, according to all related predictions. However, in the current study, researchers prefer to do experiments based on readily available data, like gender and age, even if these data are not relevant to the research question or if other researchers have already studied these data. In other words, they are barely willing to spend energy on something as laborious as data collection. It is more important for the researcher to select features based on the research question rather than the accessibility of the data. For example, Mueen et al. (2016) add some more detailed background features to their predictions, like the city of birth, transport method, and distance to the college. These features bring us opportunities to uncover the patterns behind student achievement in a richer dimension. However, the cost of collecting such detailed data is high, making it impossible to conduct big-scale experiments.

Moreover, demographics features can include any statistical factors that influence population growth or decline, which include have items, like population size, density, age structure, fecundity (birth rates), mortality (death rates), and sex ratio (Kika and Ty, 2012). However researchers claim that they use demographic features to make predictions in course failure prediction but only use gender or age. While there is nothing wrong with using the term demographic features to describe these features, a more rigorous expression is required.

#### 3.1.2. Behaviour Features

According to the classification mentioned in section 2.2.1, we review works related to learning behaviours and daily behaviours in turn.

First, the detailed recording of learning behaviour is due to the popularity of LMS and the rise of online education. Generally, an LMS is a software application for the administration, documentation, tracking, reporting, automation, and delivery of educational courses, training programs, or learning and development programs (Ellis, 2009). Features recorded by the LMS, such as clickstreams, are more detailed and indirect than traditional features. The current dramatically increased computing power contributes to the mining of the patterns behind these indirect features. Researchers focus on the LMS recorded behavioural data for prediction, based on the assumption that records in the LMS can represent certain behaviours or traits of the user. These behaviours or traits are associated with their academic performance (Conijn et al., 2016; Dominguez et al., 2016; Shruthi and Chaitra, 2016; Adejo and Connolly, 2018; Helal et al., 2018; Sandoval et al., 2018; Akçapınar et al., 2019; Liao et al., 2019; Sukhbaatar et al., 2019; Mubarak et al., 2020b; Waheed et al., 2020). Different studies are concerned with different issues. Shruthi and Chaitra (2016) collect students' behaviour data for academic performance prediction. They collected features, such as length of study, class attendance, number of library visits per week, type of books borrowed, classroom interaction, time management, and participation in extracurricular activities to make predictions. Likely, Dominguez et al. (2016) and Helal et al. (2018) also make predictions based on the data extracted from server logs of users' behaviour-based activity. Furthermore, some researchers concentrate on interactive data from online forums. Mueen et al. (2016), Azcona and Smeaton (2017), and Costa et al. (2017) all analyse student interactions in online forums and add this feature to the predictions. Ashenafi et al. (2016) design experiments that use peer-assessment data, including tasks assigned, tasks completed, questions asked, and questions answered for academically at-risk student prediction. In addition to the records left by these human activities, some implicit features are also used in predicting student academic performance. Researchers design experiments to uncover the rules behind clickstream information LMS or online learning systems. The experimental results demonstrate the validity of this feature (Aljohani et al., 2019; Liao et al., 2019; Mubarak et al., 2020b; Waheed et al., 2020). In addition to clickstream information, Li et al. (2016) record a series of student actions, such as pause, drag forward, drag back, and rate fast while watching the instructional video and add these into prediction. These implicit features do not directly reflect highly interpretable user behaviours. Still, they record all user actions at a more micro level, including laws that can be mined given the current algorithms with better fitting ability.

Moreover, besides the records left in these learning-related activities, daily behaviour-related activities are also used for predictions. Daily behaviours used in this related research generally include three categories: daily habit, internet access pattern, and social relationship. First, the popularity of smart campus cards allows students' daily habits be well recorded, such as shopping and bathing. Scholars use this data to quantify patterns of student behaviour, such as self-discipline, to analyse student performance in the course (Wang et al., 2018; Yao et al., 2019). Yao et al. (2019) use the campus card records to profile students' daily habits in three aspects: diligence, orderliness, and sleep pattern. Meanwhile, Wang et al. (2018) predict students' academic performance through features of daily habits, like daily wake-up time, daily time of return to the dormitory, daily duration spent in the dormitory, and days outside of campus. Their results demonstrate that daily habits can effectively help in the prediction of academically at-risk students. Moreover, surfing the Internet has become an integral part of college students' lives, and students surf the Internet through the campus network deployed by the school. Therefore relevant data is easily accessible. Researchers are concerned about students' Internet access patterns and find that undergraduate students' academic performance can be differentiated and predicted from their Internet usage behaviours (Zhou et al., 2018; Xu et al., 2019). Besides, social patterns are also an essential part of students' lives and have a significant impact on their performance in all aspects of their lives (Zhang et al., 2019). Gitinabard et al. (2019) use network science-related methods to analyze students' social networks and uncover their connection to their academic performance to better support struggling students early in the semester to provide timely intervention. Sapiezynski et al. (2017) also add social features to their predictions of course failure. In addition to social attributes such as degree, they also consider the impact of their friends' academic performance.

#### 3.1.3. Psychological Features

In addition to the background features and behaviours mentioned above, psychological characteristics are used to make predictions about course failure frequently (Sapiezynski et al., 2017; Ruiz et al., 2020), because research demonstrate that students' mindsets when studying determines their learning efficiency. Ruiz et al. (2020) design experiments to explore the association between students' feedback about the emotions they feel in class and their academic performance. Sapiezynski et al. (2017) collect psychological features of students through an online questionnaire and use them as features to make predictions.

However, in recent years, psychological features have not attracted much attention from researchers. The reasons are shown as follows: (1) The relationship between student psychological states and student academic performance has been well explored by researchers earlier. (2) Psychology-related data is often collected using questionnaires rather than an automated way, like an LMS log, resulting in relatively small related datasets not fitting with the big data-driven research paradigm.

As ICT advances, more data about the details of life will be recorded. This data provides us with convenience but also records a great deal of privacy. How to explore the pattern of students' daily life while avoiding privacy violations is a question worth thinking about. Moreover, researchers have mainly explored the predictability of course performance based on one of the above-mentioned categories of features, and prediction performance varies. Combining all types of features for prediction has a better chance of yielding good results.

### 3.2. Methods for Course Failure Prediction

According to the classification mentioned in section 2.2.2, we review the related works in turn.

First, as mentioned before, studies belonging to the non-deep learning type of single-model type generally aim to analyze the importance of features and the correlation between features through a white-box model, rather than pursuing predictive performance (Raut and Nichat, 2017; Saqr et al., 2017; Yassein et al., 2017). Anderton and Chivers (2016) use a generalised linear model to predict the academic performance of health science students. The results demonstrate that features like gender, course program, previous human biology, physics, and chemistry are important predictors of academic performance. Mesarić and Šebalj (2016) also use decision tree models to predict students' academic performance based on their previous academic performance. The most significant variables were total points in the state exam, points from high school, and points in the Croatian language exam. Asif et al. (2017) focus on the academic performance of different courses and try to identify courses that can serve as indicators of good or low performance at the end of the degree through a decision tree algorithm. As previously mentioned, works often attempt to explore the importance of various types of features in course failure prediction. However, the different datasets used in other studies lead to different results. A meta-analysis with sufficient rigour is required to collate experimental results in the relevant domains, to draw more convincing conclusions.

The second type is research that makes predictions based on the deep learning method. As the most popular algorithm in recent years, deep learning-related models have been widely used and have achieved good performance. Sukhbaatar et al. (2019) propose an early prediction scheme based on a deep learning model to identify students at risk of failing in a blended learning course. Waheed et al. (2020) apply a deep learning model for academic performance prediction, and experiment results show that the deep learning model outperforms statistical models like logistic regression and SVM models. Some works use and even design targeted networks to make predictions based on the patterns behind the features. Okubo et al. (2017) apply a recurrent neural network (RNN) to capture the time sequence behind log data stored in educational systems for academic performance prediction. Likely, Hu and Rangwala (2019) also make a prediction for academic performance based on the RNN model. The results demonstrate the performance of the RNN model. Mubarak et al. (2020b) apply a long short-term memory network (LSTM) (shown in Equation 8) to implicit features extracted from video clickstream data for academic performance prediction for timely intervention.


(8) $$\begin{array}{l} \begin{array}{c} i_{t}=\sigma \left(W_{xi}x_{t}+W_{hi}h_{t-1}+W_{ci}c_{t-1}+b_{i}\right)\\ f_{t}=\sigma \left(W_{xf}x_{t}+W_{hf}h_{t-1}+W_{cf}c_{t-1}+b_{f}\right)\\ c_{t}=\left(f_{t}⊙c_{t-1}+i_{t}⊙tanh\left(W_{xc}+W_{hc}h_{t-1}+b_{c}\right)\right)\\ o_{t}=\sigma \left(W_{xo}x_{t}+W_{ho}h_{t-1}+W_{co}c_{t}+b_{o}\right)\\ h_{t}=o_{t}⊙tanh\left(c_{t}\right) \end{array} \end{array}$$

where σ(*x*) is the sigmoid function defined as $\sigma \left(x\right)=\frac{1}{1+e^{-x}}$. ***W***_αβ_ denotes the weight matrix between α and β (e.g., ***W***_*xi*_ is the weight matrix from input ***x***_*t*_ to the input gate ***i***_*t*_), ***b***_α_ is the bias term of α ∈ **{***i, f, c, o***}**. Olive et al. (2019) designed several non-fully connected neural networks based on the features of the relationship between the features and achieved a good performance. In recent years a growing tendency for scholars to make predictions in this field based on deep learning techniques. Most of the works are based on existing network structures to make predictions. However, education-related data has its characteristics. It is worthwhile to consider how to create special network structures to make effective predictions based on the characteristics of educational data. Some studies that can propose their thinking about the target problem and use it to optimise the algorithm are more encouraging (Al-Luhaybi et al., 2019; Olive et al., 2019).

Moreover, many researchers choose multiple models to pursue better prediction performance. First of all, we focus on the hybrid model type research. Unlike research of the single-model type, the purpose of hybrid model type research is not to find the best performing model among the available models. Mueen et al. (2016) use three models, naive Bayes, neural network, and decision tree, to predict students' course failure, and the experiment results demonstrate that the neural network is the best model for this issue. Marbouti et al. (2016) apply seven models: logistic regression, SVM, decision tree, multi-layer perception, naive Bayes classifier, k-nearest neighbour, and an ensemble model for prediction of academic performance, separately, and find that the naive Bayes model and an ensemble model achieve the best performance. Hlosta et al. (2017) make predictions of academic performance based on a series of models, such as XGBoost, logistic regression, and SVM with different kernels, and demonstrate that XGBoost outperforms other models. Al-Luhaybi et al. (2019) propose a bootstrapped resampling approach for predicting academic performance through taking into consideration the bias issue of educational datasets. The experimental results verify the effectiveness of its algorithm. From a methodological point of view, many studies belong to the research of hybrid model type (Sandoval et al., 2018; Yu et al., 2018a; Zhou et al., 2018; Akçapınar et al., 2019; Baneres et al., 2019; Hassan et al., 2019; Hung et al., 2019; Polyzou and Karypis, 2019). The underlying logic of this type of research is that the algorithms differ in their optimization search logic and find the most suitable algorithm for course failure prediction by comparison. These researchers often conclude that a certain class of algorithms performs best on a given prediction task, whose contribution is closer to industrial applications than theoretical innovation.

Another multiple-model type is the ensemble model type, which has also attracted the attention of many researchers. Livieris et al. (2016) design experiments that allow for more precise and accurate system results for academic performance prediction. In this case, they combine the predictions of individual models utilising the voting method. The results demonstrate that no single algorithm can perform well and uniformly outperform the other algorithms. Pandey and Taruna (2016) improve the accuracy of their predictions by the same method. They combine three complementary algorithms, including decision trees, k-nearest neighbour, and aggregating one-dependence estimators. Adejo and Connolly (2018) also carry out experiments to predict student academic performance using a multi-model heterogeneous ensemble approach, and the results demonstrate the performance of ensemble algorithms. The idea of ensemble algorithms is to combine the bias or variance of these weak classifiers to create a strong classifier with better performance. In this case, most of the integrated algorithms have improved prediction results compared to the hybrid model. However, most of the current research is to integrate existing algorithms through existing integration strategies. More innovative things should be proposed, such as an ensemble strategy targeted for academic performance prediction.

From the statistical analysis to the current deep learning, the methods used by scholars in the field have evolved. The prediction results under the same experimental conditions are improved. However, most of the studies are based on existing mature algorithms for prediction. They do not improve the algorithms, making them look more like an application report than a scientific study. Further problem decomposition and algorithmic innovation based on the specified problem should be more encouraged.

## 4. Dropout Prediction

In addition to course failures, university dropouts are another issue of concern that has attracted the attention of many scholars. Exploring and predicting the patterns behind student dropouts helps schools identify education and management problems timely and improve retention rates.

The prediction for students with course failure is partly the same as the prediction for students at risk of dropping out because poor academic performance is one of the reasons why students drop out. However, in addition to course performance, many factors contribute to the prediction of students' dropout, such as personal factors, economic factors, and social features (Alban and Mauricio, 2019), which lead to the fact that this prediction is completely different from the prediction of course failure. In this case, we analyse the prediction of dropouts as a separate chapter in this paper. Note that Alban and Mauricio (2019) already did a systematic analysis of the relevant literature from 2017 and before. To avoid repetitive and meaningless work, we focus on work after 2017 and discuss their differences with the conclusions mentioned in the existing survey (Alban and Mauricio, 2019).

### 4.1. Features for Dropout Prediction

Alban and Mauricio (2019) categorise the factors that influence students' dropping out into five major categories: personal factors, academic factors, economic factors, social factors, and institutional factors.

We summarise the recent research (after 2017) based on this classification. On the one hand, some of the features used in this paper still fall into these categories mentioned in Alban and Mauricio (2019). Some studies make predictions based on previously defined features like personal information, previous education, and academic performance (Berens et al., 2018; Chen et al., 2018; Nagy and Molontay, 2018; Ortiz-Lozano et al., 2018; Del Bonifro et al., 2020; Utari et al., 2020). Moreover, some studies cover data related to the economy (Sorensen, 2019; Delen et al., 2020), which is also mentioned in the previous survey paper (Alban and Mauricio, 2019).

These studies mentioned above focus more on traditional classroom education. Regarding online education, the cost of dropping out is low due to its characteristics, leading to a more serious dropout phenomenon (Moreno-Marcos et al., 2020). In this case, an increasing number of scholars are focusing their attention on online education. The traditional education model of student learning is to complete several semesters of study and then earn a degree, making it easy to record a lot of historical data. However, in online education, many students are involved in a single course. Predicting students at risk of dropping out relies more on information generated during the learning process, i.e., learning behaviours. For example, Moreno-Marcos et al. (2020) make a prediction based on course logs and events (e.g., beginning a session, beginning a video lecture, completing a video lecture, trying an assessment, completing an assessment, etc.). Experiment results demonstrate the relationship between these features and students' dropout. Mubarak et al. (2020a) also focus on online education. They extract more diverse behavioural features from the raw data, including the average number of sessions each participant per week, the behaviour numbers of access, the number of active days per week, and so on, for dropout prediction. Some scholars have directly used implicit features hidden in the system logs to make predictions. Qiu et al. (2019) transform the original timestamp data and automatically extracts features like clickstream to predict students who are at risk of dropping out. Moreover, some studies also design models to capture the pattern behind clickstream for dropout prediction (Xing and Du, 2019; Yang et al., 2019; Goel and Goyal, 2020). In addition, some traditional education has tried to mine information generated during the learning process. Jayaraman (2020) applies data mining technologies to explore the association between students' dropouts and advisor notes, created by the student's instructor after each meeting with the student and entered into the student advising system.

As mentioned in section 3.1, if the features, such as historical academic performance and demographic features mentioned in the previous literature, are considered as static features, then information generated during the learning process is a dynamic feature. This information gives us a more flexible way to tap into the patterns behind the students. At the same time, this information contains a large amount of noise, which increases the requirements for the mining algorithm.

### 4.2. Methods for Dropout Prediction

The existing survey papers summarize the frequency of occurrence of the relevant models and the model performance (Alban and Mauricio, 2019). In this paper, we further classify the works according to the classification mentioned in section 2.2.1, and describe each category in detail.

First, works of the non-deep learning type tend to analyze the importance of features in detail along with predictions. Barbé et al. (2018) apply a basic statistical model to explore demographic features, academic performance, and social determinant factors associated with attrition at the end of the first semester of an upper-division baccalaureate nursing program. Von Hippel and Hofflinger (2020) use logistic regression to predict students who are at risk of dropping out and explore the importance of features including economic aid and major choice. Delen et al. (2020) propose a new model based on a Bayesian belief network for dropout prediction and they analyse the association of dropouts with features, including student demographic information, college matriculation features, college performance factors, scholarships, and financial support-related variables. Chen et al. (2018) propose a survival analysis framework for the early identification of students at the risk of dropping out. As can be seen, these works focus on analysing the importance of features rather than pursuing predictive performance. The other category of single-model type is the more recent and popular research based on deep learning models. Qiu et al. (2019) apply convolutional neural networks (with feature map shown in Equation 9) to predict the student dropout problem by capturing the temporal relationships behind the original timestamp data.


(9) $$\begin{array}{l} a_{j}^{l}=f\left(b_{j}^{l}+\underset{i\in M_{j}}{\sum }w_{ji}^{l}*a_{i}^{l-1}\right) \end{array}$$

where *M*_*j*_ represents the selected combination of input feature maps, and * denotes the convolution operation.$w_{ji}^{l}$ is the convolution kernel weight used for the connection between the input *i*th feature map and the output *j*th feature map in the *l*th layer. $b_{j}^{l}$ is the bias corresponding to the *j*th feature map in the *l*th layer and *f* is a nonlinear activation function, such as tanh-function or rectified linear unit (ReLU) function. Xing and Du (2019) propose to use the deep learning algorithm to construct the dropout prediction model and further calculate the predicted individual dropout probability. Muthukumar and Bhalaji (2020) predict students who are at risk of dropping out through a deep learning model with additional improvements based on temporal prediction mechanism. Mubarak et al. (2021) propose a hyper-model of convolutional neural networks and LSTM, called CONV-LSTM, for dropout prediction.

Moreover, studies of the multiple-model type also attract the attention of many scholars in dropout prediction. First of all, we review the research of hybrid model type. Del Bonifro et al. (2020) test the performance of a series of algorithms for dropout prediction, including linear discriminant analysis, SVM, and random forest. Nagy and Molontay (2018) predict dropouts by testing a wide range of models, including decision tree-based algorithms, naive Bayes, k-nearest neighbors algorithm (k-NN), linear models, and deep learning with different input settings. Pérez et al. (2018) evaluate the prediction performance of a series of models, including decision trees, logistic regression, naive Bayes and random forest, to propose the best option. Alamri et al. (2019) compare the performance of a bunch of integrated learning algorithms, including random forest, adaptive boost, XGBoost, and gradient-boost classifiers, on the prediction of students at risk of dropping out. Ahmed et al. (2020) test the performance of algorithms SVM, naive Bayes, and neural networks in predicting students at risk of dropping out, respectively. As stated in section 3.2, such studies tend to reach a more applied conclusion, and that conclusion is related to the data set they use.

Another category of multiple-model type works is ensemble model type works. Jayaraman (2020) uses natural language processing to extract the positive or negative sentiment contained in the advisor's notes and then uses the random forest model to predict student dropout. Berens et al. (2018) apply a boosting algorithm to combine multiple models, including a neural network, a regression model, and a BRF (bagging with random forest), to achieve an ensemble prediction. Moreover, Chung and Lee (2019) also chose random forest as the prediction model and achieved good performance. Chen et al. (2019) design a new ensemble model through combing a decision tree and an extreme learning machine with a single-hidden layer feedforward neural network (shown in Equation 10), and experimental results demonstrate the effectiveness of their new model.


(10) $$\begin{array}{l} \overset{L}{\underset{j=1}{\sum }}\beta_{j}g\left(w_{j}\cdot x_{i}+b_{j}\right)=o_{i},\;i=1,2,\ldots ,N \end{array}$$

where *g*(*x*) is the activation function of hidden neuron. The inner product of *w*_*j*_ and *x*_*j*_ is *w*_*j*_ · *x*_*i*_. $w_{j}={\left[w_{j1},w_{j2},\ldots ,w_{jn}\right]}^{T}$ is the weight vector of input neurons connecting to *i*_*th*_ hidden neuron. The bias of the *j*_*th*_ hidden neuron is *b*_*j*_. The weight vector of the *j*_*th*_ hidden neuron connecting to the output neurons is $\beta_{j}={\left[\beta_{j1},\beta_{j2},\ldots ,\beta_{jm}\right]}^{T}$. Generally, since the features used for prediction and the amount of data do not differ much, the methods used to predict course failure and student dropout are relatively similar.

## 5. Mental Health Problems Detection

In this section, we review works related to the detection of mental health problems. Although many universities have set up counseling facilities to help students with mental health problems, not every student with mental health problems will come forward to seek help. Timely detection of students with mental health problems not only helps prevent extreme behaviours such as self-harm but also provides a reference for studying the causes behind students' psychological problems. Unlike the first two parts, all works related to mental health problems detection are biased towards analyzing the importance of features in detection rather than pursuing detection performance. Note that different studies have various concerns. Some works focus on depression, while others focus on self-harm and suicidal behaviour. In this paper, we focus on research methodology design at the level of features and methods rather than detailed meta-analysis. Therefore, we do not analyse the knowledge involving the professional part of psychology.

### 5.1. Features for Mental Health Problems Detection

Three types of features are frequently mentioned in related works: background features, psychology-related features, and life experiences.

First, background features, especially demographic features, are used to make detections about mental health problems frequently. Shannon et al. (2019) use demographic features to detect student-athlete and non-athlete intentions to self-manage their mental health. Kiekens et al. (2019) use demographic features to detect the incidence of non-suicidal self-injury in college students. Stewart et al. (2019) apply demographic features to the detection of mental health help-seeking orientations. Ebert et al. (2019) detect the major depressive disorder onset of college students through demographic features. A large number of relevant studies has proved the relationship between demographic features and psychology.

The second and most studied category is features of psychology-related fields, such as historical, psychological test results, indicators of specific psychological dimensions, and life-specific experiences. Chang et al. (2017) design to test the role of ethnic identity and loneliness in predicting suicide risk in Latino college students. Maguire et al. (2017) apply emotional intelligence to the prediction of cognitive and affective engagement in higher education. Cassady et al. (2019) predict student expression based on general and academic anxieties. Ge et al. (2020) use historical psychometric records to predict psychological states among Chinese undergraduate students in the COVID-19 epidemic.

The relationship between unique life experiences and the students' psychological state has also attracted the attention of a large number of scholars. Ebert et al. (2019) add features related to childhood-adolescent traumatic experiences into the prediction of major depressive disorder. Odacı and Çelik (2020) apply traumatic childhood experiences to the prediction of the disposition to risk-taking and aggression in Turkish university students. Kiekens et al. (2019) also use traumatic experiences to predict the incidence of non-suicidal self-injury in college students. Brackman et al. (2016) focus on the prediction of suicidal behaviour and study the association between non-suicidal self-injury and interpersonal psychological theories of suicidal behaviour with suicidal behaviour, respectively.

The two types of features, psychological and lived experience features. These experiences are often collected through questionnaires, which are expensive to administer and difficult to promote on a large scale. How to infer these features indirectly through easily available data is a research direction worth exploring.

### 5.2. Methods for Mental Health Problems Detection

At the methodological level, works related to detecting students with psychological anomalies have mainly pursued the correlations behind the variables rather than the prediction accuracy. In this case, all works in this part belong to the non-deep learning type in the single-model type according to the classification mentioned in section 2.2.2. Scholars in this field prefer to apply white box models with high explainability. Some works prefer to apply regression-based models to explore the laws behind the variables. Babaei et al. (2016) apply multiple regression to evaluate the importance of metacognition beliefs and general health for alexithymia. Likely, Chang et al. (2017) use multiple regression to examine the role of ethnicity, identity and loneliness as predictors of suicide risk. Stewart et al. (2019) explore the association between stigma, gender, age, psychology coursework, and mental health help-seeking orientations, respectively, through logistic regression analysis. Meanwhile, Al-Shahrani et al. (2020) collect socio-demographic and academic features of students through a self-administered questionnaire and apply logistic regression to analyze the relationship behind them. Moreover, Cassady et al. (2019) apply hierarchical regression to examine the role of general and academic anxieties in the detection of students' depression. In general, the regression-based algorithm is a relatively traditional algorithm for exploring relationships between variables. Several scholars have also explored this problem of predicting students with psychological problems using recent machine learning white-box models. Ge et al. (2020) apply XGBoost to predict depression in college students in the COVID-19 epidemic.

Deep learning techniques are also widely used in the prediction of psychological problems (Yang et al., 2017; Squarcina et al., 2020; Wang et al., 2020), but the scope of their research is not for students. Detection for students at risk of psychological problems is manually collected data rather than data automatically generated by information systems or ICT devices, like student behaviours in the learning process and campus life.

## 6. Prediction of Difficulty in Graduation

In this section, we review the works related to the students with difficulty in graduation, including the following two categories: students who can not meet the graduation requirements and students who can not graduate on time. Note that there is a fundamental difference between a student who can not meet graduation requirements and a student who can not graduate on time. The former may be a dropout, while the latter will defer graduation. Predicting these students can help identify the students who are at risk of graduation. Thus, management can intervene timely and take essential steps to train the students to improve their performance.

### 6.1. Features for Predicting Difficulty in Graduation

The relevant works are reviewed in terms of features. Generally, the features used in these works mainly are belonged to background features, including demographic features and historical academic features.

First, as an important feature, academic performance has received a lot of attention in related fields. Some studies prefer to predict student graduation based on academically relevant features. Pang et al. (2017) predict students' graduation based on their historical grades over multiple semesters. Ojha et al. (2017) and Tampakas et al. (2018) use demographic features and historical academic features to predict students' graduation time. Andreswari et al. (2019) introduce more detailed background information. In addition to demographic features and historical academic features, they also add parents' jobs and income for graduation time prediction, and the results demonstrate the effectiveness of these features. Moreover, Hutt et al. (2018) focus on 4-year college graduation and add academic performance into its prediction. Qin and Phillips (2019) looked at whether students will graduate early (3 years to graduation) and added academic features to the projections for that category of students. Adekitan and Salau (2019) provide a more detailed breakdown of the difficulty in graduation: graduation with poor results or may not graduate at all, and predicts it through academic features. Yu et al. (2018b) focus on the graduation issues for students with learning disabilities and predict them with academic features.

Overall the features used in the relevant predictions for difficulty in graduation are not rich enough. The possible reason is that graduation-related predictions have not received enough attention due to the inaccessibility of data. High-quality public datasets should be published to encourage scholars to explore this direction.

### 6.2. Methods for Predicting Difficulty in Graduation

According to the classification in section 2.2.2, all works in the related field are also divided into two categories: single-model type and multiple-model types, including four sub-categories: non-deep learning type, deep learning type, hybrid model type, and ensemble model type.

First, as mentioned before, research on non-deep learning single model types tends to explore the impact of features on predictive targets through white-box models, like logistic regression models, decision trees, naive Bayes classifiers, and so on. Gershenfeld et al. (2016) explore the importance of first semester grades in graduation prediction through a logistic regression model. Likely, Yu et al. (2018b) also apply logistic regression model to analyse the importance of high school academic preparation and postsecondary academic support services for the prediction of college completion among students with learning disabilities. Andreswari et al. (2019) use C4.5 algorithms, a type of decision tree algorithm, to explore the relationship between student graduation, and academic performance, and family factors like parents' jobs and income. Purnamasari et al. (2019) also use C4.5 algorithms to explore how academic performance can impact the final graduation time of students. Kurniawan et al. (2020) develop a graduation prediction system based on C4.5 algorithms. Meiriza et al. (2020) leverage naive Bbayes classifier to analyse the influence of demographic features and academic performance on college graduation. Due to the small amount of relevant research data, no researcher has attempted to predict by deep learning algorithms for the time being.

Second, to pursue prediction performance, researchers choose to use multiple models for their predictions. First of all, we summarise the research that belongs to the hybrid model type. Ojha et al. (2017) apply three models for employment prediction, including SVM, gaussian processes, and deep Boltzmann machines, and test their performance separately. Tampakas et al. (2018) design a two-level classification algorithm framework. By comparing with the Bayesian model, multi-layer perceptron, integrated algorithm, and decision tree algorithm, they prove the advantages of the proposed framework in student graduation prediction. Wirawan et al. (2019) design experiments to predict the timeliness of graduation through the C4.5 algorithm, naive Bayes, and k-NN. As mentioned before, this kind of research has more application value than theoretical innovation. Meanwhile, meta-analysis is necessary in order to draw a reliable conclusion. Moreover, other researchers have chosen ensemble learning algorithms to predict students at risk of graduation. Pang et al. (2017), and Hutt et al. (2018) predict graduation-related problems with the random forest algorithm and ensemble SVM algorithm, respectively.

## 7. Prediction for Difficulty in Employment

Employment is the top priority for college students. Finding a good job can help students succeed, otherwise, it will have a negative impact on their lives. Accurate employment predictions and targeted interventions in advance are effective ways to solve this problem. In this case, scholars explore to predict the future employment situation of students through machine learning.

### 7.1. Features for Predicting Difficulty in Employment

Researchers in this field tend to make predictions based on background features, including historical academic performance and demographics features. Li and Zhang (2020), Zhou et al. (2020), and He et al. (2021) use detailed and rich background features to predict student employment, including academic achievement, scholarship, graduation qualification, family status (whether poor or not), and association member and so on. Moreover, Li et al. (2018) add occupational personality data based on historical academic performance to predict student employment. Gershenfeld et al. (2016) focus on the earliest indicators of academic performance-first-semester grade point average and attempts to use it to predict students' employment upon graduation.

Some studies try to dig out more detailed features hidden behind the features. Guo et al. (2020) is concerned with student employment issues and predicts the employment of students taking into account employment bias. Unlike other works that use GPA to quantify academic performance, they propose a novel method that overcomes the heterogeneity in student performance by using one-hot encoding + autoencoder (Equation 11) to obtain a more valid representation of student performance.


(11) $$\begin{array}{l} \begin{array}{c} h_{\left(2\right)}=f\left(W_{\left(2\right)}h_{\left(1\right)}+b_{\left(2\right)}\right)\\ h_{\left(3\right)}=f\left(W_{\left(3\right)}h_{\left(2\right)}+b_{\left(3\right)}\right)\\ h_{\left(i\right)}=f\left(W_{\left(i\right)}h_{\left(i-1\right)}+b_{\left(i\right)}\right),i=1,2,...k \end{array} \end{array}$$

where *f* is the activation function and ***W***_(*i*)_, ***b***_(*i*)_ are the transformation matrix and the bias vector.

It makes sense that academic features would be used as the main predictor of students' academic performance. However, students study a variety of subjects, and each subject corresponds to a type of knowledge. Each type of work requires specific knowledge rather than all the knowledge learned. For example, algorithm engineers and front-end engineers are both computer science majors. The former places more emphasis on mathematics and data structures, while the latter emphasises programming skills related to code. However, current research quantifies academic performance as a whole (like GPA) rather than quantifying specific knowledge individually. Although Guo et al. (2020) propose a valid representation of academic achievement by representing learning to overcome the disadvantage of losing information distribution of GPA. But the unexplainability of its results does not entirely solve the problem.

### 7.2. Methods for Predicting Difficulty in Employment

Although the number of related studies is not large, the methods used are varied. Both Li and Zhang (2020) and Zhou et al. (2020) apply C4.5 decision tree for student employment prediction. Gershenfeld et al. (2016) apply a set of logistic regression models to mine the relationship between first-semester GPA and students' employment. They are all belonged to the single model type. Moreover, Guo et al. (2020) chose to use deep learning to predict student employment. They design a deep-learning-based framework, including autoencoder and LSTM with special dropout (Equation 12), for students' employment prediction.


(12) $$\begin{array}{l} \begin{array}{c} i_{t}=\sigma \left(W_{xi}x_{t}+W_{hi}h_{t-1}+W_{ci}c_{t-1}+b_{i}\right)⊙m_{i}\\ f_{t}=\sigma \left(W_{xf}x_{t}+W_{hf}h_{t-1}+W_{cf}c_{t-1}+b_{f}\right)⊙m_{f}\\ c_{t}=\left(f_{t}⊙c_{t-1}+i_{t}⊙tanh\left(W_{xc}+W_{hc}h_{t-1}+b_{c}\right)\right)\\ ⊙m_{c}\\ o_{t}=\sigma \left(W_{xo}x_{t}+W_{ho}h_{t-1}+W_{co}c_{t}+b_{o}\right)⊙m_{o}\\ h_{t}=o_{t}⊙tanh\left(c_{t}\right)⊙m_{h} \end{array} \end{array}$$

where ⊙ represents element-wise product and ***m***_*f*_, ***m***_*c*_, ***m***_*o*_, and ***m***_*h*_ are dropout binary mask vectors, with an element value of 0 indicating that dropout happens, for input gates, forget gates, cells, and output gates, respectively.

Moreover, some works belong to the multiple-model type. Mishra et al. (2016) apply several models, like bayesian methods, multilayer perceptrons, and sequential minimal optimization, ensemble methods and decision trees, for employability prediction, separately to find the best one. He et al. (2021) predict students' employment through a random forest model.

The importance of research on employment prediction is self-evident. The current challenges in this field are dataset. Unlike the difficulty of collecting psychology-related data, employment-related administrative departments, such as related companies and government departments, store a large amount of data. Then, the phenomenon of data islands is so serious that it is difficult for researchers to obtain relevant data.

## 8. Challenges

Despite the considerable efforts of the scholars involved, challenges still exist. In this section, we analyse the current challenges in this field from the following four perspectives: data, privacy, dynamics, and explainability.

### 8.1. Data

In a data-driven era, the quality of the dataset guarantees the reliability of the experimental results. The size of the dataset has a significant impact on the quality and credibility of the experimental results. In general, experimental results based on larger datasets are more likely to avoid bias in the data and be trustworthy. Meanwhile, experiments to compare and validate the performance of the algorithm need to be based on the same dataset. For example, there are well-known high-quality datasets such as the MNIST dataset, MS-COCO dataset in the field of computer vision, and IMDb film comment dataset and Twenty Newsgroups dataset in the field of natural language processing. In this case, high-quality public datasets need to be proposed. This allows researchers to compare the performance of algorithms in the same environment and thus get more reliable and trustworthy results. However, there are currently no recognised public datasets in the field of educational anomaly analytics.

### 8.2. Privacy

It is well-known that privacy and big data are in an adversarial relationship at this stage. At the theoretical level, the more data, the more reliable the experimental result. However, compared to other areas, privacy in education is more sensitive because it concerns students who are physically and mentally immature (Yu et al., 2021a). Relevant data collectors and the systems cannot adopt the principle of ‘the more the better’ to collect all personal data of students roughly. On the contrary, the data collection work needs to proceed cautiously according to the specific needs of the relevant tasks. Meanwhile, this has resulted in a very limited number of high-quality public datasets related to education, compared with other fields, like natural language processing and computer vision. How to build trust between data holders and data analysts is the most important way to solve this problem. Currently, related workers are trying to solve this problem from two aspects: 1. Improving education- and big data-related laws to clarify the relationship and responsibilities of all participants so as to build trust through legal constraints. 2. Promote federated learning, data sandbox, and other related techniques to segregate data while training algorithms to secure privacy.

### 8.3. Dynamics

Predictions for educational anomalies are time-sensitive. The earlier an accurate prediction is made, the better the chances of making an effective intervention. For example, if a prediction of a student's future employment can be made in the first semester of college, the probability of its effective intervention is much higher than if it is made in the fourth-semester (Gershenfeld et al., 2016). However, fewer studies in related fields have mentioned the analysis of dynamics.

### 8.4. Explainability

As mentioned before, in recent years, increasing scholars have used deep learning to predict students' abnormal behaviour. While these scholars achieved better prediction results, they also brought the biggest drawback of current deep learning-unexplainability. This can easily cause educators to mistrust the prediction (Al-Doulat et al., 2020), and thus affect the diffusion and application of the technology in the industry.

## 9. Conclusion

In this survey, we systematically summarise research on educational anomaly analytics. We focus on five types of research that received the most attention, including course failure prediction, dropout prediction, mental health problems detection, prediction of difficulty in graduation, and prediction of difficulty in employment. For each type of problem, we analysed the overall educational anomalies in terms of the features used, and the prediction method. Finally, we discussed the challenges of existing studies in this field.

Overall, scholars in the educational anomaly analytics field are very active in introducing machine learning methods. However, they tend to use existing algorithms directly rather than develop new ones. Compared with other fields, educational anomaly analytics field has its own challenges and requirements. In addition to the challenges we highlighted in section 8, the development of ICT (Information and Communication Technology) will make it possible to collect more diverse educational data, which provides a more severe challenge for education big data mining. Existing general algorithms cannot cope with these challenges, and targeted algorithms need to be developed to effectively mine educational data. This requires close cooperation between education researchers and machine learning researchers. Moreover, the research of educational anomaly analytics is closely related to real-world applications. Encouraging the development of related education management systems and getting feedback from practical applications are effective means to promote the development of related fields.

## Author Contributions

TG, XB, and FX contributed to conception and design of the study. All authors contributed to manuscript writing and revision, read, and approved the submitted version.

## Conflict of Interest

The authors declare that the research was conducted in the absence of any commercial or financial relationships that could be construed as a potential conflict of interest. The handling editor declared a past co-authorship with one of the authors FX.

## Publisher's Note

All claims expressed in this article are solely those of the authors and do not necessarily represent those of their affiliated organizations, or those of the publisher, the editors and the reviewers. Any product that may be evaluated in this article, or claim that may be made by its manufacturer, is not guaranteed or endorsed by the publisher.
